# Improvements in 2D p-type WSe_2_ transistors towards ultimate CMOS scaling

**DOI:** 10.1038/s41598-023-30317-4

**Published:** 2023-02-27

**Authors:** Naim Hossain Patoary, Jing Xie, Guantong Zhou, Fahad Al Mamun, Mohammed Sayyad, Sefaattin Tongay, Ivan Sanchez Esqueda

**Affiliations:** 1 Electrical, Computer and Energy Engineering, Arizona State University, Tempe, AZ 85281 USA; 2 Materials Science and Engineering, School for Engineering of Matter, Transport and Energy, Arizona State University, Tempe, AZ 85287 USA

**Keywords:** Electronic devices, Electrical and electronic engineering, Electronic devices

## Abstract

This paper provides comprehensive experimental analysis relating to improvements in the two-dimensional (2D) p-type metal–oxide–semiconductor (PMOS) field effect transistors (FETs) by pure van der Waals (vdW) contacts on few-layer tungsten diselenide (WSe_2_) with high-k metal gate (HKMG) stacks. Our analysis shows that standard metallization techniques (e.g., e-beam evaporation at moderate pressure ~ 10^–5^ torr) results in significant Fermi-level pinning, but Schottky barrier heights (SBH) remain small (< 100 meV) when using high work function metals (e.g., Pt or Pd). Temperature-dependent analysis uncovers a more dominant contribution to contact resistance from the channel access region and confirms significant improvement through less damaging metallization techniques (i.e., reduced scattering) combined with strongly scaled HKMG stacks (enhanced carrier density). A clean contact/channel interface is achieved through high-vacuum evaporation and temperature-controlled stepped deposition providing large improvements in contact resistance. Our study reports low contact resistance of 5.7 kΩ-µm, with on-state currents of ~ 97 µA/µm and subthreshold swing of ~ 140 mV/dec in FETs with channel lengths of 400 nm. Furthermore, theoretical analysis using a Landauer transport ballistic model for WSe_2_ SB-FETs elucidates the prospects of nanoscale 2D PMOS FETs indicating high-performance (excellent on-state current vs subthreshold swing benchmarks) towards the ultimate CMOS scaling limit.

## Introduction

**Figure 1:**
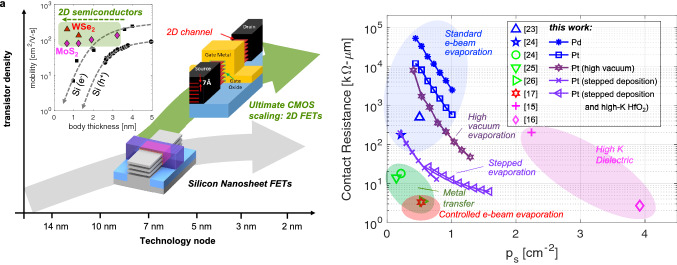
(**a**) Roadmap for introduction of 2D materials in CMOS technology to enhance scaling, density of integration, and chip performance, as well as to enable new functionality (e.g., in CMOS + X), and 3D monolithic integration. Two tracks are shown, one for silicon gate-all-around (GAA) FETs with stacked nanosheets (each sheet ~ 5 nm in thickness), and one for FETs with stacked 2D nanosheets (< 1 nm thickness for monolayer semiconductors) © [2021] IEEE. Reprinted, with permission, from^15^. Inset shows mobility degradation with scaling the both thickness in Si devices (e.g., the nanosheet thickness), while 2D semiconductors shown negligible degradation down to a monolayer. (**b**) Summary of trends on improvements of 2D PMOS FET contact resistance as function of sheet carrier density and the methods to achieve those improvements. Results from this work show advances in contact resistance from standard e-beam evaporation to high-vacuum and stepped evaporation on devices with scaled high-K dielectric and metal gate (HKMG) stacks.

Two-dimensional (2D) materials have gained significant interest for their application in electronic devices, circuits, and systems, since the discovery of graphene in 2004^1^. Semiconducting 2D transition metal dichalcogenides (TMDs) are promising candidates to enable continued downscaling of field-effect-transistors (FETs) supporting Moore’s law for years to come^2^. This is due to their layered van der Waals (vdW) structure that can be thinned down to a single atomic sheet (< 1 nm) with dangling-bond-free surfaces. A thin semiconducting body is needed for extreme scaling of transistors to maintain good electrostatic control of the channel thereby suppressing short-channel effects (SCE) that would otherwise degrade their performance. In conventional CMOS technology, bulk semiconductors are used to construct the channel, but these cannot be scaled below ~ 5 nm in thickness without introducing significant challenges related to variation, surface roughness, and dangling bonds, which lead to the degradation of charge carrier mobility (see inset Fig. 1a)^2–4^. This presents a limit to the scaling of transistors (e.g., cannot be scaled to channel lengths below ~ 10 nm), even for non-planar designs such as FinFET and nanowire/nanosheet devices. However, sub-10 nm channel length FETs with well-controlled electrostatics and suitable mobilities can be fabricated using 2D semiconductors (e.g., TMDs) which retain their desirable electronic properties even at the limit of a single atomic layer (i.e., monolayer with thickness < 1 nm)^5–9^. Thus, 2D semiconductors may be integrated into CMOS processes to enable the ultimate scaling of CMOS technology. Additionally, 2D FETs can be integrated in the back-end-of-line (BEOL) of CMOS processes to support monolithic 3D integration of integrated circuits (ICs) based on multi-tier integration of 2D devices^10^. Moreover, different 2D materials can be stacked to assemble unique artificial materials known as van der Waals (vdW) heterostructures that may enable new and improved electronic functionality^11–13^. Figure 1a illustrates a roadmap for the introduction of 2D semiconductors in CMOS technology to enhance density of integration and improve chip performance, as well as for monolithic 3D integration and added functionality (e.g., CMOS + X)^14^. A state-of-the-art silicon gate-all-around (GAA) FET with stacked “nanosheets” (~ 5 nm thickness) is shown along the silicon track, whereas an FET with stacked 2D nanosheets (< 1 nm thickness) illustrates the ultimate scaling of the same device architecture along the 2D materials track^15^. The inset shows the effect of body thickness scaling on carrier mobility, contrasting the prohibitive degradation in silicon^3,4^ against 2D semiconductors (MoS_2_, WSe_2_)^5–9^ showing little or no effect in mobility down to a monolayer (< 1 nm).

CMOS technology uses complementary n-type and p-type transistors to implement logic functions. To achieve the ultimate scaling of CMOS technology with 2D materials, both n-type and p-type MOS (i.e., 2D NMOS and 2D PMOS) FETs are needed. While substantial efforts have been dedicated to improving 2D NMOS devices (e.g., using MoS_2_ semiconducting channels), the performance of 2D PMOS FETs has fallen behind. Consequently, new efforts have emerged^15–17^ to elucidate the weaknesses of 2D PMOS devices and to develop improved methods to enhance their performance. In the typical device configuration, metal contacts are placed directly over the 2D semiconducting channel, forming Schottky barriers (SB), and are referred to as SB-FETs^18–21^. As established by previous work^6,19^, this leads to the contacts playing a significant role in the operation and performance of the device. However, a comprehensive study of contacts in 2D PMOS FETs in the context of these recent efforts is lacking. Therefore, this paper presents an updated analysis of 2D WSe_2_ PMOS devices with emphasis on contact resistance and its role in device performance. Moreover, recent work has shown that strongly scaled insulators are needed to enhance gate control and reduce the impact of the Schottky barriers^22^. Still, previous efforts aimed at improving 2D PMOS FETs through improved contacts have only demonstrated devices without scaled insulators (i.e., used silicon substrate as common gate with thick gate dielectrics > 100 nm)^17^. In contrast, our analysis is based on SB-FETs with few nm high-K metal-gate (HKMG) stacks and demonstrates various methods aimed at improving contact resistance. Furthermore, we report improvements in 2D PMOS FET performance benchmarks against the best published results, and elucidate the performance limits of extremely scaled 2D PMOS FETs through quasi-ballistic transport models based on the Landauer formalism.

Figure 1b summarizes the results from this work and other recent efforts^15–17,23–26^ aimed at lowering contact resistance in 2D WSe_2_ PMOS FETs. This figure plots contact resistance as a function of carrier concentration in the semiconducting channel (carriers are holes for PMOS devices and their density increases as we push the device deeper into strong inversion). As shown, standard methods used to deposit contacts, such as electron-beam (e-beam) evaporation, typically leads to large contact resistance (blue shaded region). However, significant improvements can be obtained by either metal transfer^24–26^ or through a controlled e-beam evaporation^17^ (green and red shaded regions respectively). Also, larger carrier concentrations can be achieved using high-K dielectrics in the gate stack (improved gate capacitance), and this may also alleviate contact resistance^15,16^ (pink shaded region). Our experimental results indicate a progression in contact resistance improvement starting from standard e-beam evaporation (pressure ~ 10^–5^ torr), followed by high vacuum evaporation (pressure ~ 10^–6^ torr), then higher vacuum (~ 10^–7^ torr) and stepped deposition. As previously discussed, a high vacuum metal deposition helps minimize damage to the underlying channel and improves resistance^5^. Moreover, a stepped evaporation can help maintain the sample near room temperature also reducing damage. Finally, by switching from Al_2_O_3_ to HfO_2_ (high-K of ~ 17) we achieve further improvements. These are further explained in the Discussion section of this paper.

## Results

### Device fabrication

**Figure 2:**
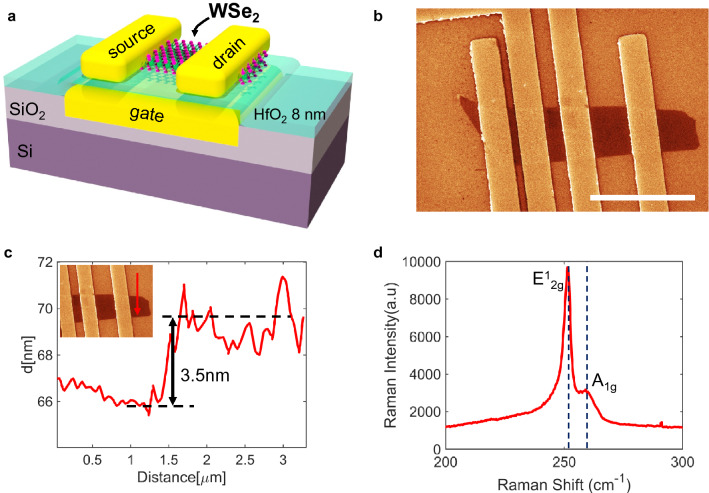
(**a**) Schematic of 2D WSe_2_ PMOS FETs studied in this work. The FETs are configured in a transfer length method (TLM) structure and have a gate-first design with high-K metal-gate (HKMG) stack. Two different gate dielectrics were used in this work (Al_2_O_3_ and HfO_2_) with a thickness of 8 nm. See “Methods” for fabrication details. (**b**) Scanning electron microscope (SEM) image of the WSe_2_ TLM structure (scale bar indicates 3 µm in length). (**c**) Atomic force microscopy (AFM) surface topography scan (non-contact mode) reveals a WSe_2_ channel thickness of ~ 3.5 nm (approximately 5 layers). (**d**) Raman spectrum of the channel region showing E^1^_2g_ and A_1g_ peaks consistent with ~ 5 layers of WSe_2_.

P-type conduction in 2D FETs can be achieved using a variety of techniques, including contact engineering, chemical doping, and/or electrostatic doping. Our work uses a transfer length method (TLM) structure to demonstrate p-type devices with few layer (3–5 layer) WSe_2_ channels. These are designed and fabricated with a high-K metal-gate (HKMG) stack featuring 8 nm of either Al_2_O_3_ or HfO_2_ gate dielectrics. High work function (WF) metal contacts were used to facilitate the injection and conduction of positively charged carriers (holes) into the WSe_2_ channels. This is enabled through the suitable alignment of metal contact Fermi levels with the edge of the valence band in the semiconducting channel. This work presents results on devices with Pt (WF of 5.65 eV)^27^ and Pd (WF of 5.22 eV)^17^ metal contacts. A 3D schematic of the TLM structures is shown in Fig. 2a. In brief, a gate-first technique was used to fabricate FETs with HKMG stacks and various channel lengths on a TLM configuration (See “Methods” and Supplementary Fig. 1 for fabrication details). As shown by previous work^28^, the gate-first approach can lead to improved 2D FET uniformity, channel mobility, and subthreshold swing. Our gate-first HKMG process includes patterning and deposition of a Cr/Au metal gate (e-beam lithography, evaporation, and lift-off) followed by atomic-layer deposition (ALD) of the high-K dielectric (8 nm Al_2_O_3_ or HfO_2_). The thickness and relative permittivity of the ALD dielectrics are verified by non-contact atomic force microscopy (AFM) and capacitance measurements (see Supplementary Figs. 2 and 3). Once the gate stack is in place, a deterministic transfer process is used to form the WSe_2_ channel. Here, exfoliated WSe_2_ samples were carefully selected through optical inspection to obtain the desired thickness and homogeneity across the entire TLM structure. After transferring the WSe_2_ channels, the metal contacts (i.e., the source and drain electrodes of the FETs) are prepared through e-beam lithography, deposition, and lift-off. We note that the quality of the contacts, and consequently the performance of the FETs, depends strongly on this metal deposition process as will be described in the sections below. The contacts are placed at different distances to result in FETs with various channel lengths. A scanning electron microscopy (SEM) image of a typical sample is show in in Fig. 2b. The SEM images provide verification of the surface morphology and WSe_2_ channel uniformity. Additional analysis and precise determination of the channel thickness is obtained from atomic force microscopy (AFM) surface topography scans across the WSe_2_ regions. A typical AFM measurements is shown in Fig. 2c where a step in the surface profile reveals a channel thickness of ~ 3.5 nm (this corresponds to approximately 5 layers of WSe_2_). To further verify the quality and number of WSe_2_ layers Raman spectroscopy in the channel region of the fully fabricated FETs have been conducted. A typical Raman spectrum is shown in Fig. 2d, where the first and second peaks, centered near 250 cm^−1^, correspond to the E^1^_2g_ and A^1^_g_ modes. As explained in previous work^29^, these vibrational modes and the position of the peaks are sensitive to the number of WSe_2_ layers. For example, the E^1^_2g_ peak will experience a right shift (away from 250 cm^−1^) with increasing number of layers, but the A^1^_g_ mode exhibits little dependency on film thickness. In our Raman spectra we observe right shifts in E^1^_2g_ peaks that are consistent with approximately 4–5 layers of WSe_2_. In addition to the peak position, the full width at half maximum (FWHM) can serve to indicate the crystalline quality of the WSe_2_ sample^30^. We observe FWHM of ~ 4.8 cm^−1^ indicating that the crystalline quality of the channel regions has not been compromised after the fabrication process. See “Methods” for details on SEM, AFM, and Raman measurements.

### Electrical characterization

**Figure 3:**
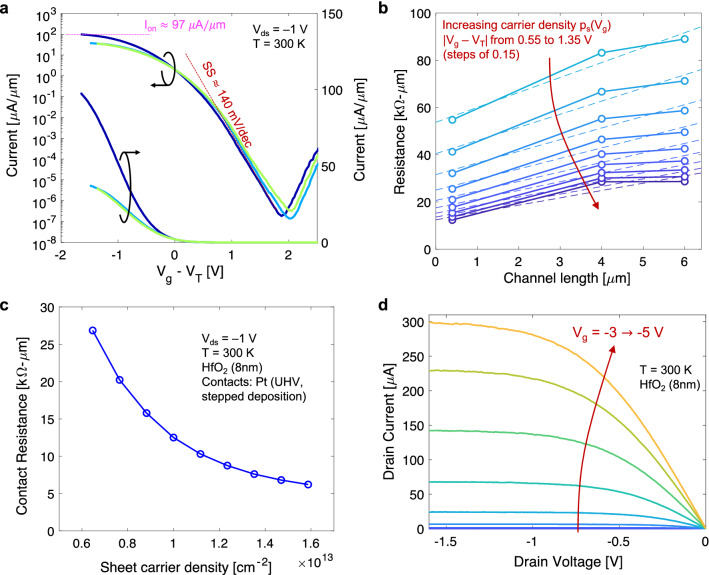
(**a**) Drain current (I_d_) vs. gate voltage above threshold (V_g_–V_T_) for WSe_2_ PMOS FETs with different channel lengths (400 nm, 4 µm, 6 µm) measured at room temperature with drain-to-source voltage of V_ds_ = – 1 V. These devices show a subthreshold swing of ~ 140 mV/dec and on-state current normalized to channel width (W) of about 97 µA/µm **(b)** Total resistance vs. channel length for increasing sheet carrier density (as labelled, measurements correspond to V_g_–V_T_ ranging from – 0.55 V to – 1.35 V). (**c**) Contact resistance extracted from extrapolation to L = 0 as a function of channel sheet carrier density. (**d**) Drain current (I_d_) vs. drain voltage (V_d_) characteristics under different gate biasing conditions from subthreshold to strong inversion (V_g_ in steps of – 0.25 V).

Electrical measurements of the 2D PMOS devices is used to extract key performance parameters such as on-state current per micrometer of channel width (W), the inverse subthreshold slope (i.e., the subthreshold swing), the contact resistance, and the channel mobility. Typical drain current (I_d_) versus gate voltage (V_g_) characteristics measured at room temperature (T = 300 K) for three different channel lengths (L = 400 nm, 4000 nm, and 6000 nm) are shown in Fig. 3a (see “Methods” for details on electrical characterization). The I_d_–V_g_ characteristics are measured with a drain-to-source voltage of V_ds_ = – 1 V and are plotted in both linear and logarithmic scale for the current axis (y-axis). Minimal to negligible gate leakage was observed for all device (see Supplementary Fig. 4). These measurements are from devices with 8 nm HfO_2_ gate dielectric and with Pt/Au (10 nm/25 nm) source and drain contacts deposited under high vacuum (10^–7^ torr) using a stepped evaporation approach (I_d_–V_g_ measurements for devices with Al_2_O_3_ gate dielectric are shown in Supplementary Fig. 5). This high vacuum and stepped evaporation approach resulted in the best performing devices (see “Discussion” for more details). As labeled in Fig. 3a, for a device with L = 400 nm we obtain on-state current I_on_ $\approx$ 97 µA/µm (normalized to W), and subthreshold swing SS $\approx$ 140 mV/dec. These values are indicative of high-performance 2D FETs and are further analyzed in context of recent published results in the “Discussion” section below.

As mentioned earlier, contacts play a significant role in the performance of these 2D SB-FETs and necessitates further investigation. Using the transfer length method (TLM) we analyze measurements of resistance (obtained from normalized I_on_) as a function of L to extract contact resistance and hole mobility respectively from the vertical intercept (i.e., extrapolation to L = 0) and from the slope^31,32^. Figure 3b plots resistance (in units of kΩ-µm) as a function of channel length for various levels of channel sheet carrier density given by p_s_ = (1/q)C_ox_(|V_g_ – V_T_|), where C_ox_ is the oxide capacitance per unit area and V_T_ is the threshold voltage. Thus, a larger p_s_ corresponds to a larger gate bias above threshold and results in lower contact resistance (vertical intercept). Here, V_T_ was extracted at a fixed I_d_ = 10 μA. Similar results in the analysis of resistance are obtained using V_T_ extracted from the extrapolation of a linearly fit to the I_d_-V_g_ data at the peak transconductance (g_m_). Figure 3c plots the contact resistance as a function of sheet carrier density reaching a value as low as ~ 6 kΩ-µm. This is comparable to the recently reported record PMOS contact resistance of 2.7 kΩ-µm, and to other recently reported value of 3.3 kΩ-µm but at smaller sheet carrier densities^16,17^ (both of these previous results, along with our own results shown in Fig. 1b). As described below (see “Discussion”), a similar contact resistance at a smaller sheet carrier density may be indicative of better quality interface between metal contact and semiconducting channel. For our own devices, which combine the methods of these previous efforts (i.e., a low pressure stepped evaporation and a HKMG stack), we expect further reduction in contact resistance with reduced base pressure during metal evaporation (currently limited to ~ 10^–7^ torr with our existing tools). Nonetheless, these WSe_2_ PMOS FETs already show outstanding performance with superior off-state performance (as indicated by SS) and cutting-edge on-current levels (as indicated by I_on_) compared to previous work (see “Discussion”). We note that hole mobilities extracted from the slope of the resistance vs. channel length in Fig. 3b ranges from approximately 125 up to ~ 150 cm^2^/V-s (see Supplementary Fig. 6). Further discussion of device performance and benchmarking of I_on_ vs SS is provided below. Additionally, the supporting information (see Supplementary Fig. 6) shows the extraction of sheet resistance as a function of carrier density. For completion, Fig. 3d plots I_d_ as a function of V_d_ at different gate biasing conditions ranging from the off-state (V_g_ < V_T_) to on-state (V_g_ > V_T_). The I_d_–V_d_ family of curves in Fig. 3d are from room temperature measurements on 2D WSe_2_ PMOS FETs with 8 nm HfO_2_ gate dielectric and a channel length L = 400 nm. The gate voltage was changed from – 3 to – 5 V in steps of – 0.25 V.

### Analysis of Schottky barriers

**Figure 4:**
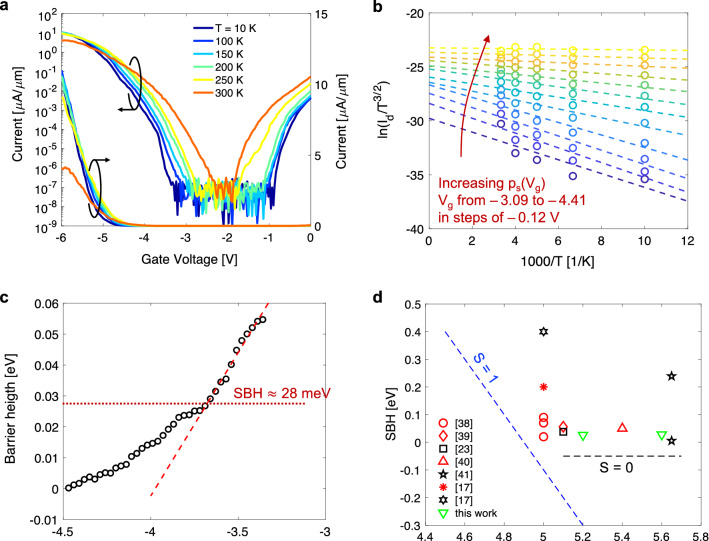
(**a**) I_d_–V_g_ characteristics at different temperatures ranging from 300 K down to 10 K for WSe_2_ PMOS FET with L = 400 nm, and for V_ds_ = –1 V. Current (normalized to channel width) is plotted in linear (right axis) and logarithmic scale (left axis). (**b**) Arrhenius plot of thermionic emission current in 2D semiconductor obtained for different values of V_g_ (from – 3.09 to – 4.41 V) corresponding to different values of sheet carrier density, p_s_. The slope (dashed lines) can be used to extract barrier height. (**c**) Extractions of barrier height plotted as a function of gate bias, the dashed lines indicate the flat-band voltage for which a deviation from the linear trend is observed, and where barrier height is exactly the Schottky barrier height. (**d**) Our extracted Schottky barrier heights (SBH) as a function of the metal work function. Also shown are extractions from previous published works on WSe_2_ PMOS FETs with different types of metals. The collection of experimental results appear to indicate significant Fermi level pinning (S ~ 0). However, the SBH is small (< 100 meV) in most cases, suggesting other factors contributing to large contact resistivity observed in 2D PMOS FETs.

The Schottky junctions between source/drain contacts and the semiconducting channel play a crucial role in device performance as these can significantly impact contact resistivity^6,19^. A critical parameter for these junctions is the Schottky barrier height (SBH), which indicates the potential energy barrier obstructing the injection of charge carriers from metal to semiconductor. We can select metal contacts with WF that result in good band alignment (i.e., a small SBH) and ideally a seamless injection of charge carriers into the channel. However, non-ideal effects may be at play, such as Fermi-level “pinning”, affecting our ability to correctly adjust the SBH^33,34^. Therefore, it is essential to extract the SBH to identify the contribution of Schottky junctions on contact resistivity. Figure 4a plots the I_d_–V_g_ characteristics for a WSe_2_ PMOS FET with Al_2_O_3_ gate dielectric, Pt/Au contacts (deposited with standard e-beam evaporation technique), and L = 400 nm, measured at various temperatures from 300 K down to 10 K, and V_ds_ = – 1 V. We note that a different I_on_ is achieved in this device (compared to Fig. 3a) because of a larger contact resistance resulting from the standard e-beam evaporation technique. However, it is still useful to extract SBH from the off-state region measurements of this device. In the off-state region of operation (V_g_ >  ~ –4 V in this device), source-to-drain conduction is limited by thermionic emission of charged carriers over large energy barriers at the metal/semiconductor interface. For this thermally activated process, a higher temperature results in more charge injection into the channel and larger current as evidenced in Fig. 4a.

The theory of thermionic emission for 2D semiconductors dictates that the current is given by ${\text{I = WA}}^{*} {\text{T}}^{3/2}{\text{exp}}\left( {- \frac{{{\text{q}}_{{\phi _{B}}}}}{{{\text{k}}_{{\text{B}}} {\text{T}}}}} \right)\left[ {{1} - {\mkern 1mu}{\text{exp}}\left( {- \frac{{{\text{qV}}_{{{\text{ds}}}}}}{{{\text{k}}_{{\text{B}}}{\text{T}}}}}\right)}\right]$, where A^*^ = q(8πk_B_^3^m^*^)^1/2^/h^2^ is the Richardson constant, k_B_ is the Boltzmann constant, h is Planck’s constant, and ϕ_B_ is the barrier height^35–37^. Considering the equation for thermionic emission, we can extract barrier height (ϕ_B_) from the slope of ${\text{Ln}}(\text{I/}{\text{T}}^{3/2})$ as a function of $1/\text{T}$ as shown in Fig. 4b for different values of V_g_. The extractions of barrier heigh for different V_g_ are plotted in Fig. 4c. As V_g_ is increased negatively towards the on-state, the height of the potential energy barrier drops down linearly. At flat-band, further change in V_g_ will not continue to reduce the barrier height. Instead, a deviation from the linear trend is observed in the extractions with increasing V_g_, as the barrier becomes narrower and tunneling start to contribute significantly to the injection of carriers into the channel. The transition is labeled in Fig. 4c at a flat-band voltage of approximately – 3.7 V. At this voltage, the extracted barrier heigh corresponds to the Schottky barrier height (SBH), for which we obtain ~ 28 meV for Pt contacts, and ~ 25 meV for Pd contacts (experimental I–V at various temperatures and extractions of SBH for Pd contacts in Supplementary Fig. 8). The extractions of SBH are plotted in Fig. 4d as a function of metal work function (WF). This plots includes our experimental results, as well as extractions of SBH from previous works on WSe_2_ using different metal contacts^17,23,38–41^. A blue dotted line labeled with slope S = 1 illustrates the ideal case at the Schottky–Mott limit^42^, where a change in WF translates directly to a chance in SBH. However, the collection of experimental results seem to reveal a weak dependence of SBH on WF (S ~ 0), indicative of severe Fermi-level pinning. While this could be a concern for contact resistivity, the SBH is still small (< 100 meV) in most cases, suggesting that other mechanisms could be responsible for the large contact resistivity typically observed in 2D PMOS FETs. In fact, our work shows that significantly different contact resistance can be achieved in devices with the same metal WF for which we extract similar low SBH (see Fig. 1b). Indeed, the access resistance (i.e., the resistance of the semiconducting channel underneath the metal contact) can have a more dominant contribution towards the contact resistance if significant damage is introduced in this region during the metal deposition. Accordingly, we attribute the large improvements in contact resistance achieved in our WSe_2_ PMOS FETs to reduced damage in the semiconducting channel access regions (underneath the metal contact) through adjustments in the deposition process (e.g., high-vacuum evaporation), and by implementing a stepped evaporation/deposition approach. As discussed in previous work, these changes can help minimize damage in the access regions resulting in overall improvements in contact resistivity and FET performance^5,17^.

## Discussion

**Figure 5:**
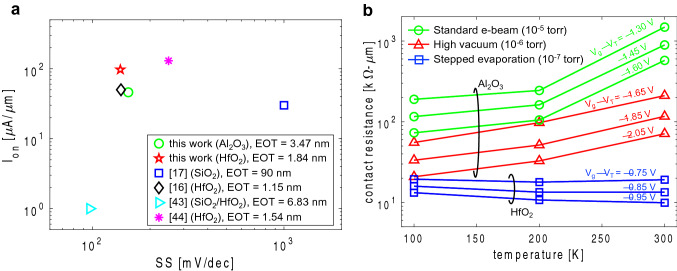
(**a**) 2D PMOS FET performance benchmark of on-state current (I_on_) versus subthreshold swing (SS). This plot includes our results from WSe_2_ devices prepared with high-vacuum evaporation and stepped metal contact deposition (low contact resistivity of ~ 6 kΩ-µm) with two different high-K dielectrics (Al_2_O_3_ and HfO_2_). Also shown are previous results with record contact resistivities. Our devices show improvements indicated by higher I_on_ while maintaining low SS (see text). (**b**) Contact Resistance as a function of temperature from three devices prepared with different metal evaporation techniques. For each case three curves are shown corresponding to three different V_g_ (i.e., different sheet carrier density). Moving from standard e-beam evaporation (10^–5^ torr), to mid-level vacuum (10^–6^ torr), and higher vacuum (10^–7^ torr) plus stepped evaporation we see a reduction in contact resistance and a transition in the temperature dependence (see text).

We have shown the electrical characteristics of high-performance WSe_2_ PMOS FETs and have demonstrated significant improvements in contact resistivity through high-vacuum evaporation and stepped deposition of metal contacts while using HKMG stacks. Here we show benchmarking of on-state current (I_on_) versus subthreshold swing (SS) against previous work to indicate superior FET performance. Figure 5a compares our experimental results for devices prepared using low base pressure evaporation and stepped deposition of the metal contacts (to achieve low contact resistivity of ~ 6 kΩ-µm, comparable to previous efforts) against previous reports^16,17,43,44^. For comparison, the effective oxide thickness (EOT), calculated as t_ox_*(K_SiO2_/K_ox_), is labeled for each data point. Here, t_ox_ and K_ox_ are the thickness and dielectric constant of the gate oxide, and K_SiO2_ is the dielectric constant of SiO_2_. Our results are for devices with 8 nm Al_2_O_3_ as well as 8 nm HfO_2_ gate dielectrics (I_on_ extracted at sheet carrier densities of ~ 7 × 10^12^ and ~ 1.6 × 10^13^ cm^−2^ respectively) with few layer (~ 3 layers) of WSe_2_. Compared to previous reports, we show improvements in performance as determined by a large I_on_ while simultaneously a small SS. We note, however, that our data is on devices with few layers (~ 3 layers) of WSe_2_ compared to other efforts which are devices with WSe_2_ monolayer channels. Nonetheless, our results indicate that significant improvements in contact resistivity combined with scaled HKMG architectures can result in desirable improvements in 2D PMOS FETs towards ultimate scaling of CMOS technology.

As already established, contacts play a crucial role in the performance and operation of 2D FETs, and the Schottky junctions at the metal connections with the channel are a key part of this role. However, our results show that even under significant Fermi-level pinning (i.e., poor control of Schottky barrier height with metal work function), the SBH for hole injection into WSe_2_ channels is typically small (< 100 meV). Furthermore, contact resistance appears to be more strongly dependent on the methods used to evaporate and deposit the metal contacts, and less by the workfunction of the metal and the SBH. Thus, we determine that resistance of the channel access region (i.e., the WSe_2_ region underneath the metal contact) can contribute significantly to contact resistance^5,45^. By using methods to fabricate metal contacts that minimize the damage to the access region we reduce the overall contact resistivity and improve performance. As further evidence we explore the temperature dependence of contact resistance for devices fabricated using different methods (i.e., standard e-beam evaporation, evaporation at lower pressure, and further reduction in pressure plus stepped deposition). Figure 5b plots contact resistance as a function of temperature for all three devices at different levels of sheet carrier density (i.e., different V_g_, with larger V_g_ resulting in larger sheet carrier density and smaller contact resistance). The results show a clear transition from contact resistance *increasing* with temperature (i.e., dominated by scattering in the access region) for devices fabricated using standard e-beam evaporation at 10^–5^ torr, to contact resistance *decreasing* with temperature (i.e., dominated by thermionic emission in the Schottky barriers) for devices fabricated using a stepped evaporation at lower pressures of 10^–7^ torr^5^. By using a high vacuum and stepped evaporation, less damage is introduced during fabrication of the contacts leading to reduced scattering in the access region. Moreover, a high-K gate dielectric (~ 17 for HfO_2_) under the access region may enhance carrier density also reducing the access resistance. Altogether, this helps to significantly improve (reduce) the contact resistance to where we start to see a transition in the temperature dependence (dominance of Schottky barriers or interface resistance). This interface vs access resistance effects were previously observed in MoS_2_ n-channel FETs.

**Figure 6:**
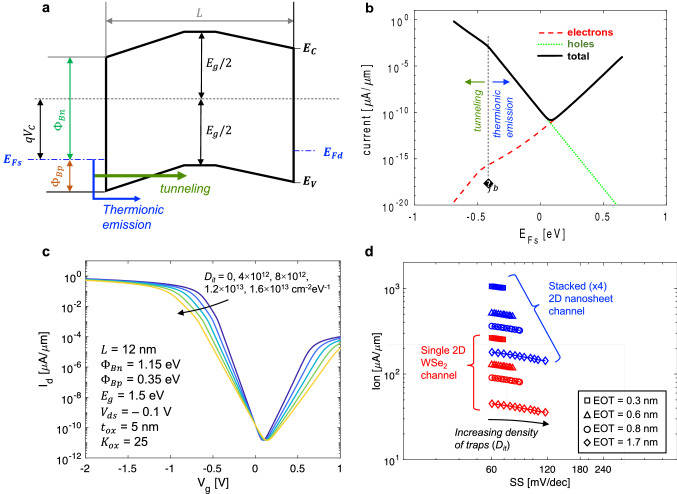
(**a**) Energy-band diagram for short-channel 2D WSe_2_ PMOS FET with definitions of relevant energy levels and potentials used in calculation of ballistic FET I-V characteristics (see text). (**b**) Calculations of electron, hole, and total FET drain-to-source current as a function of the channel potential (i.e., relative position of the Fermi-level in the source to the semiconductor bands). Note that the zero energy reference is exactly at the middle of the bandgap. (**c**) Calculation of ballistic drain-to-source current as a function of V_g_ for various levels of interface trap density (model parameters labelled in this plot). (**d**) Model predictions of I_on_ vs SS in ballistic devices with different gate dielectrics (thickness and dielectric constant) as well as different levels of interface trap density. All values are for V_g_ = – 2 V (sheet carrier density vary from ~ 10^12^ to ~ 7 × 10^12^ cm^−2^). Results in red are for single 2D WSe_2_ channel, results in blue are for stacked 2D nanosheet channels (2D nanosheet FETs, see Fig. 1a).

Finally, we elucidate the performance limits of extremely scaled 2D PMOS FETs using a ballistic transport model based on the Landauer formalism^46–48^. Here, the drain-to-source current for is given by $\text{I = (2q/h)}\int \text{dE T(E) M}\left({\text{E}}\right)\text{ [}{\text{f}}_{\text{S}} \, - \, {\text{f}}_{\text{D}}\text{]}$, where (2q/h) represents the quantum of conductance, M(E) is the density of modes in the 2D channel, T(E) is the transmission coefficient, and f_S_(f_D_) is the Fermi function in the source(drain). For a ballistic device the transmission coefficient in the channel is equal to one (no backscattering), but T(E) account for thermionic emission and tunneling through the Schottky barriers at the contacts. Tunneling probabilities are based on the WKB approximation and assume triangular barriers (see Fig. 6a)^18,21,49–52^. The application of a gate voltage adjusts the energy bands in the 2D semiconductor, thus modulating the Schottky barriers (height and width) for injecting carriers into the channel. This effect is captured in the model based on the capacitive coupling of the gate to the channel as given by ${\text{V}}_{{\text{C}}} = \left( {{\text{V}}_{{\text{g}}} - {\text{V}}_{0} } \right)\frac{{{\text{C}}_{{{\text{ox}}}} }}{{{\text{C}}_{{{\text{ox}}}} + {\text{C}}_{{\text{q}}} \left( {{\text{V}}_{{\text{C}}} } \right)}}$, where ${\text{C}}_{\text{q}}$ is the quantum capacitance of the channel, ${\text{C}}_{\text{ox}}$ is the oxide capacitance, and ${\text{V}}_{0}$ is the gate voltage for which the channel potential is zero (i.e., for which the source Fermi-level align with middle of the gap). The parameter ${\text{V}}_{0}$ allows accounting for a metal–semiconductor work function difference and for the effects of surface states (interface traps)^20,21,53,54^. More details of the modeling approach are provided in the supplementary information. Figure 6b plots the current contribution from holes (green dotted line) in the valence band, electrons (red dashed line) in the conduction band, as well as the total current (solid black line) as a function of the channel potential. The labels in the plot show where the I–V characteristics transition from purely thermionic emission to where tunneling starts to contribute (this happens when bands go flat at the metal/semiconductor junctions). Figure 6c plots the drain current vs gate voltage (I_d_–V_g_) characteristics at room temperature for interface traps densities (D_it_) of 0, 4 × 10^12^, 8 × 10^12^, 1.2 × 10^13^, and 1.6 × 10^13^ cm^−2^ eV^−1^. We note that these are typical trap densities for FETs with 2D channel materials^22^. Model parameters for these calculations are shown in Fig. 6c. As shown, interface traps can impact on-state current as well as subthreshold swing. The calculations in Fig. 6b serve only as an example of the modeling approach and the model parameters were not adjusted to fit any particular device. Instead, we now apply the Landauer-based model to simulate ballistic WSe_2_ SB-FETs (SBH adjusted to fit extractions from WSe_2_ FETs with Pt contacts) and estimate performance benchmarks of I_on_ vs SS in nanoscale devices with ballistic transport. We show calculations for devices with ultrascaled gate dielectrics with different EOT, as well as for different levels of interface trap density. Figure 6d plots the calculations of I_on_ vs SS in single channel 2D WSe_2_ FETs (red lines with symbols), as well as for stacked 2D nanosheet FETs (blue). All values are for V_g_ = – 2 V (sheet carrier densities vary from ~ 10^12^ to ~ 7 × 10^12^ cm^−2^). The model calculations indicate that large I_on_ (~ 270 µA/µm) values can be achieve together with ideal SS values (~ 60 mV/dec) in single-channel device with 2 nm HfO_2_ gate dielectrics at moderate sheet carrier densities of ~ 7 × 10^12^ cm^−2^ (larger I_on_ can be achieved at higher sheet carrier densities by further scaling of t_ox_, or at larger V_g_). Moreover, by stacking 2D nanosheets a multiplicative effect on current will result in significant enhancement in performance.

This work provides significant insights on emerging methods to enhance contact quality and improve performance of FETs with 2D channel materials. This is presented in context of recent efforts that have emerged to elucidate the issues encountered with 2D PMOS devices. Comprehensive analysis is given for characterizing contact resistance, and to identify the key components that contribute to contact resistance in 2D PMOS FETs. Devices prepared using these improved methods, such as high-vacuum evaporation and stepped deposition of metal contacts to reduce damage to channel access regions, as well as using scaled HKMG stacks, resulted in noticeable improvements on benchmarks of I_on_ vs SS. Moreover, we present a physics-based model estimates for nanoscale ballistic WSe_2_ devices to elucidate the performance of extremely scaled 2D PMOS FETs towards the ultimate CMOS scaling limits.

## Methods

### TLM device fabrication

90 nm Si/SiO_2_ Substrate has been patterned using the EVG Aligner 620 photolithography with a chrome shadow mask. Substrate was prepatterned with independent back gate by etching 40 nm into Si/SiO_2_ and depositing metal layer, followed by liftoff. An ALD layer of 8 nm Al_2_O_3_ or HfO_2_ was deposited using Cambridge Savannah ALD Deposition Tool at 180 °C. ALD film quality was checked using non-contact mode AFM surface topography scanning. Few layer WSe_2_ was obtained by mechanical exfoliation of commercially available Tungsten bulk crystal using the adhesive plastic film supplied by Ultron technology. Visual assessment of the optical contrast on TMD flakes on the PDMS resulted in the selection of WSe_2_ flake with rectangular shape with uniform thickness. Selected flake was deterministically transferred using a custom-built transfer station at SAES lab on top of the ALD on trench gated region. Electron beam lithography was conducted with JEOL 6000FS to define the TLM pattern of source and drain electrodes using PMMA(Polymethyl methacrylate) as mask. followed by developed in IPA and MIBK (3:1) developer solution at room temperature. The metals (Pt/Au, Pd/Au) were deposited by E-beam metal evaporation (Lesker 3) at a rate of 2 Armstrong per second at a pressure of 10^–6^ torr to ensure the uniformity of the electrode pads. Acetone was used to do liftoff to obtain the final TLM FET structure with a defined channel length of 400 nm, 800 nm and 1.8 µm. The approximate thickness of WSe_2_ flakes used in this study are 3 ~ 5 atomic layer as verified by tapping mode atomic force microscopy (AFM) and Raman Spectroscopy. Stepped e-beam evaporation process, for instance, included several steps of 10 nm Pt depositions at a rate of 2 Å/s. After each 10 nm of deposition, the evaporation was stopped for an hour to allow the material to cool and return to room temperature.

### Room-temperature Raman

Room temperature micro Raman spectroscopy was carried out in a Janis ST-500. Using a specially constructed Raman spectrometer with a 180° geometry, the Raman data were gathered. A 150 mW Coherent Sapphire SF laser with a 532 nm laser wavelength was used to stimulate the sample. A neutral density filters wheel was used to adjust the laser power, which started at 100mW.

### Atomic force microscopy measurements

The surface morphology and thickness is characterized by a XE-100 modular AFM. PSIA 910 M-NCHR probe with an 8 nm tip radius is used to record the sample height in a non-contact mode and 256-line resolution. The images are plotted and processed using Gwyddion software.

### Scanning electron microscopy measurements

The surface topography is characterized by a Hitachi S-4700 Field Emission Scanning Electron Microscope (SEM). A variable beam accelerating voltage of 0.5–30 keV was used to capture the detail image.

### Electrical measurements

The electrical measurements both room and low temperature was conducted on a Lakeshore semi-automatic probe station using a Keysight b1500a parameter analyzer system. The in situ temperature controlled I–V measurements were performed using three of the source measure units (SMUs) connected to Lakeshore Cryotronics probestation 8425 at a vacuum of 10^–6^ torr with ZN50R-CVT probe (uninterrupted variable temperature probes) connected to the SMUs. The data were derived directly as a function of applied voltage for different temperature. Temperature was read using a temperature controller M336 with a chart recorder LSA16S2 (Kelvin vs time).

## Supplementary Information

Supplementary Information.

## Data Availability

Data that supports the plots within this paper and other findings of this study are available from the corresponding author upon reasonable request.
