# Supplementary material for: Improvements in 2D p-type WSe2 transistors towards ultimate CMOS scaling
Source: Sci Rep. 2023 Feb 27;13:3304. doi: 10.1038/s41598-023-30317-4 (PMC9971212; doi:10.1038/s41598-023-30317-4)
Supplement: Supplementary file 1 — Supplementary Information. [file 41598_2023_30317_MOESM1_ESM.pdf]

Supplementary Information

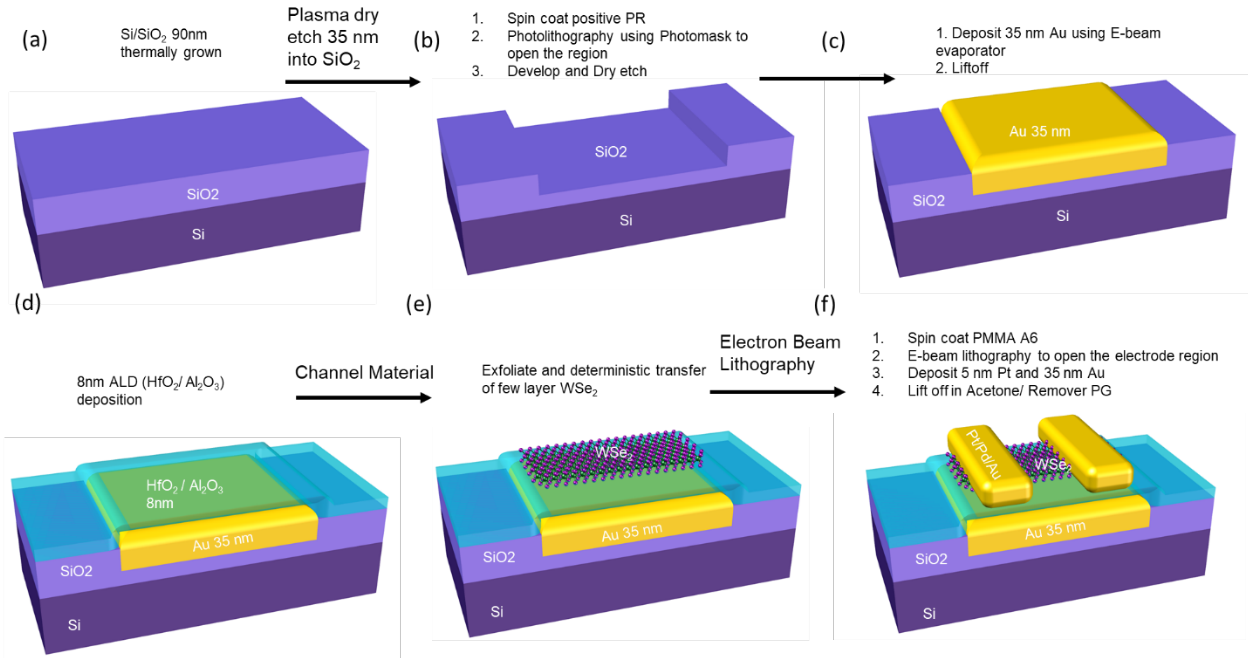

Supplementary Figure 1. (a)3D schematic of Si/SiO2(90 nm) (b) Si/SiO2 has been trenched by oxygen plasma at a rate of 60 Å per second. (c) E-beam evaporation of gate metal (d) 8 nm ALD at 180 ºC (e) Exfoliate and deterministic transfer of few-layer WSe2. (f) Electron beam lithography to pattern source and drain electrode, followed by metal evaporation and liftoff.

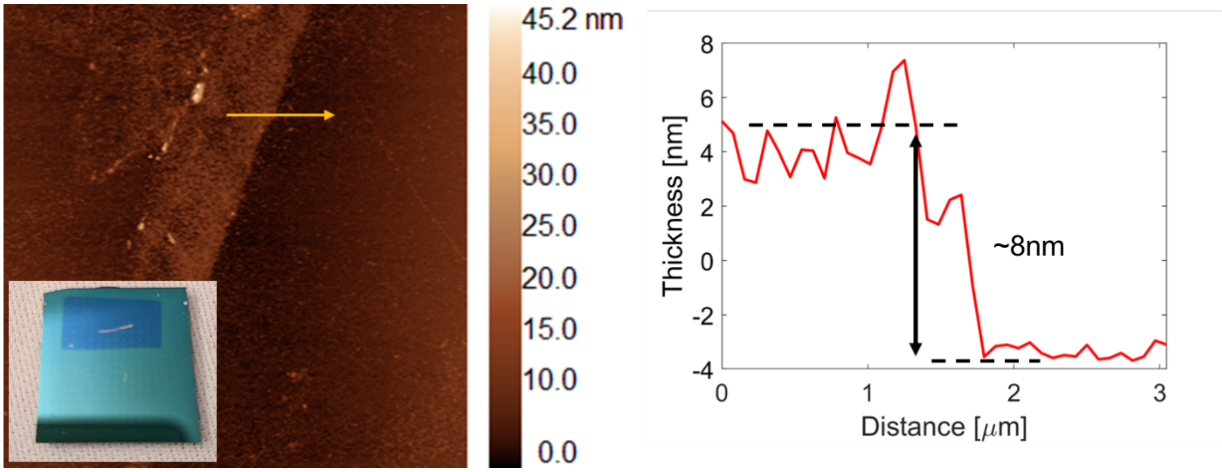

Supplementary Figure 2. Atomic force microscopy (AFM) surface topography (left) indicating a step at the edge of the ALD HfO2.The step height is approximately 8 nm as labeled in the line profile (right). This is the thickness of the gate dielectric in the gate-first

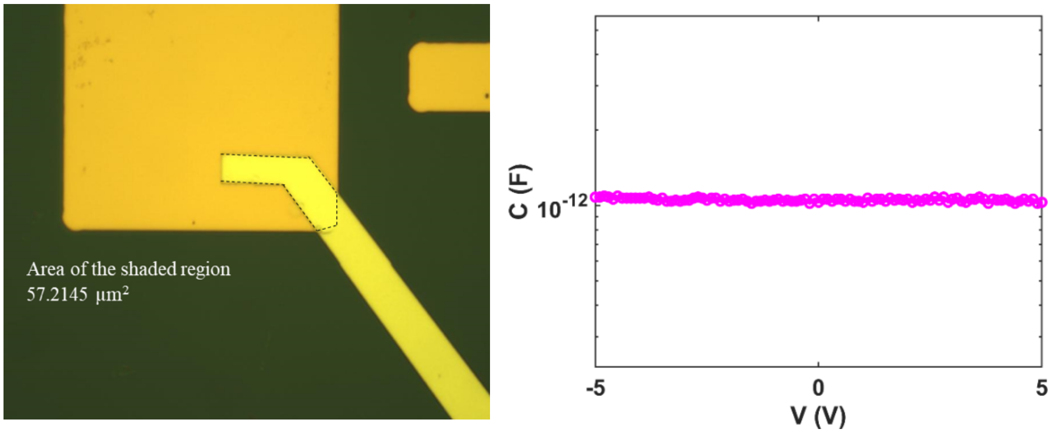

Supplementary Figure 3. (left) Optical microscope image showing the metal-insulator-metal (MIM) capacitance test structure (i.e., a parallel-plate capacitor with 8 nm HfO_2 ALD dielectric). The area of the capacitor (enclosed by dashed line) is ~57.2 µm^2. (right) The capacitance is measured at ~1 pF over the full range of relevant voltages. The dielectric constant of the oxide is obtained as $K_{ox} \;=\; Ct_{ox}/\varepsilon_0 A$, where C is capacitance, A is the area, ε_0 is the permittivity of free space (8.854 x 10^{-12} Fm^{-1}) and t_{ox} = 8 nm is the oxide thickness. We extract a dielectric constant of 16.889 for HfO_2.

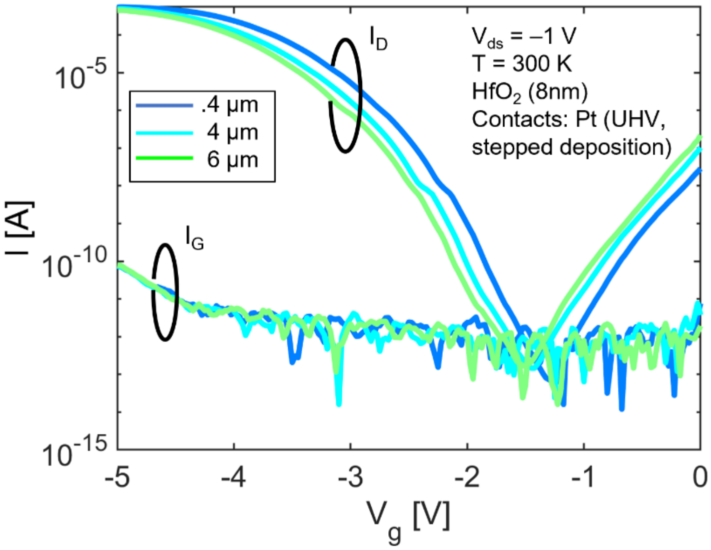

Supplementary Figure 4. For WSe_2 FET with stepped (10^{-7} torr) metal (Pt) evaporation and HfO_2 8nm as the gate stack, drain current (I_d) and gate current (I_g) as a function of gate voltage (V_g) for various channel lengths have been presented. As shown, the gate leakage is negligible (measurements at noise floor and orders of magnitude below I_d) over the entire range of relevant V_g.

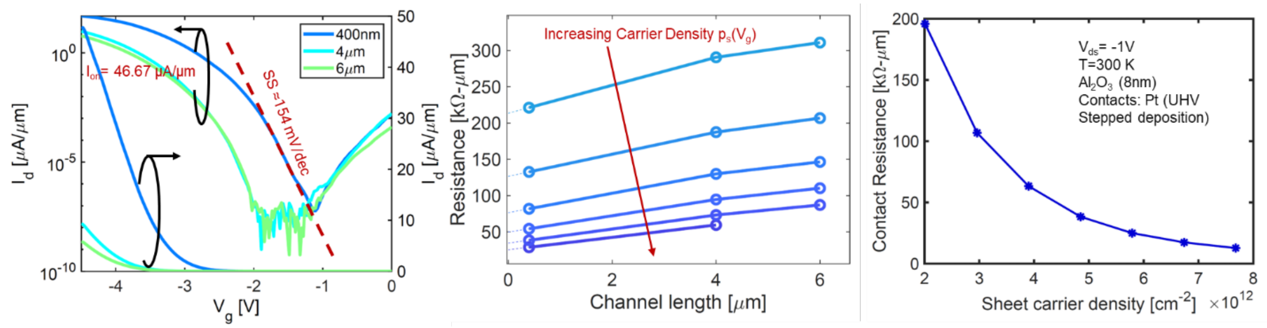

Supplementary Figure 5. WSe_2 FET with stepped high vacuum (10^{-7} torr) metal (Pt) evaporation with Al_2O_3 8nm as gate stack (a) I_d-V_g as a function of temperature for WSe_2 PMOS FETs with different channel lengths (400 nm, 4 μm, 6 μm) measured at room temperature with drain-to-source voltage of V_{ds} = –1 V. (b) Total resistance vs channel length for increasing sheet career density. (c) Contact resistance as a function of Sheet Carrier density

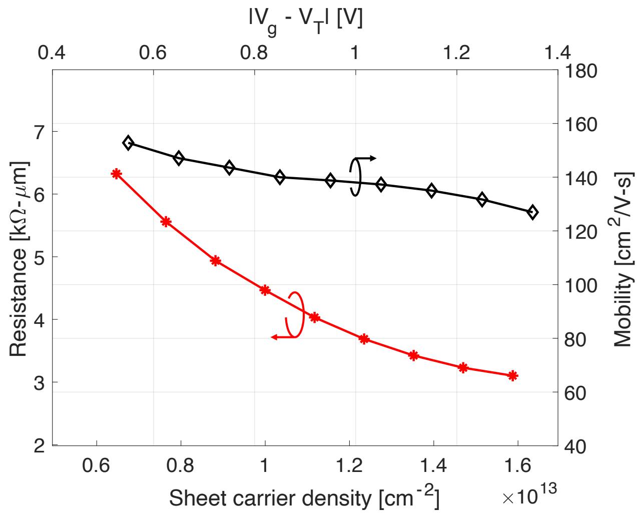

Supplementary Figure 6. Sheet resistance and field effect mobility has been extracted as a function of sheet carrier density. Sheet resistance and field effect mobility have both been extracted using the slope of resistance normalized by width for various channel lengths.

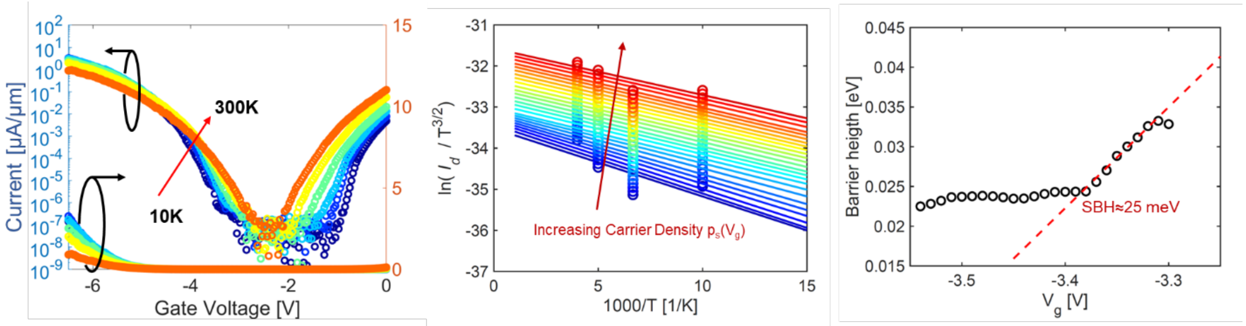

Supplementary Figure 7. WSe_2 FET with standard (10^{-5} torr) metal (Pd) evaporation with Al_2O_3 8nm as gate stack (a) I_d-V_g as a function of temperature for WSe_2 PMOS FETs with 400 nm channel lengths with drain-to-source voltage of V_{ds} = –1 V. (b) Thermionic emission current in a 2D semiconductor as plotted by Arrhenius for various values of Vg (different values of sheet carrier density, p_s). The slope (dashed lines) is used to extract barrier height. (c) Extractions of barrier height plotted as a function of gate bias, The flat-band voltage for which a break from the linear trend is seen is indicated by the dashed lines., and where barrier height is exactly the Schottky barrier height

Modeling Approach:

The modeling methodology follows a Landauer-transport approach for SB-MOSFETs presented by Sanchez Esqueda[1]. This approach calculates current using the Landauer equation given by

$$I = \frac{2q}{h}\int_{-\infty}^{\infty} T(E)M(E)[f(E,E_{Fs}) - f(E,E_{Fd})]dE,$$

where f is the Fermi function, $M(E)$ is the density of modes (e.g., $M(E) = (g_v/\pi h)[2m_h^*(E_V - E)]^{1/2}$ in the valence band, where g_v is the valley degeneracy and m_h^* is the hole effective mass). The transmission coefficient $T(E)$ is obtained based on the series combination of scatterers and is given by[2]

$$T = \left[1 + \left(\frac{1 - T_S}{T_S}\right) + \left(\frac{1 - T_D}{T_D}\right) + \left(\frac{1 - T_C}{T_C}\right)\right]^{-1}.$$

For energies between the edge of the conduction band and the peak of the barrier for holes or electrons, T_S and T_D are calculated using the WKB approximation for tunneling probabilities across a triangular shaped barrier as[3]

$$T_{WKB} = exp\left\{-\frac{2\pi}{h}\int_0^{x_0} \sqrt{2m_h^*[E - E_V(x)]dx}\right\},$$

and T_C (i.e., transmission through the channel) is set equal to 1 for ballistic transport. The Fermi level at the source/drain is respectively given by $E_{Fs} = qV_C + qV_{ds}/2$ and $E_{Fd} = qV_C - qV_{ds}/2$. The relationship between the potential in the channel (V_C) and V_{gs} is determined by capacitive coupling of the gate to the channel[4–6] and is calculated as

$$V_C = (V_g - V_0)\frac{C_{ox}}{C_{ox} + C_q(V_C)},$$

where C_q is the quantum capacitance of the channel given by

$$C_q(V_C) = \frac{q^2}{4k_BT_L}\int_{-\infty}^{+\infty} D(E)sech^2\left(\frac{E - V_C}{2k_BT_L}\right)dE,$$

and $D(E)$ is the density of states in the channel (containing both conduction and valence bands)[4]. Here, V_0 accounts for the work-function difference between the gate and the channel (Φ_{MS}) and contains the charge contribution from interface traps. Interface traps can be acceptor-like or donor-like and trap occupancy is calculated using Fermi functions[6,7]. The resulting expression for V_0 is given by

$$V_0(V_C) = \Phi_{MS} - \frac{q}{C_{ox}}\left\{\int_{-\infty}^{+\infty} D_{it,a}(E)f(E,qV_C)dE - \int_{-\infty}^{+\infty} D_{it,d}(E)[1 - f(E,qV_C)]dE\right\},$$

where $D_{it,a}(E)$ and $D_{it,d}(E)$ are the acceptor and donor-like interface trap densities respectively. The transcendental equation for V_C must be solved numerically to obtain a self-consistent solution. Supplementary Figure 4 shows an example of the channel potential calculated as a function of the gate voltage for different densities of interface traps.

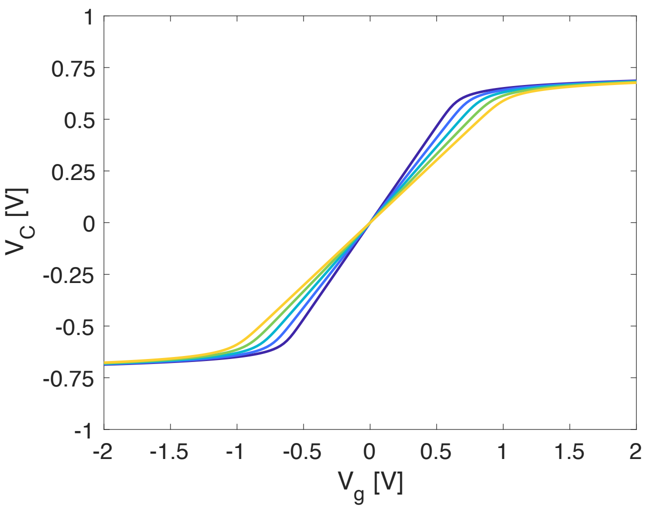

Supplementary Figure 8. Calculation of channel potential as a function of gate voltage for various levels of interface trap density (model parameters same as those in main text Figure 6c). The calculations include both acceptor-like and donor-like traps with densities ranging from 10^{12} up to 10^{13} $cm^{-2}eV^{-1}$

References

1. Esqueda, Ivan S., Tian, He, Yan, Xiaodong & Wang, Han. Transport Properties and Device Prospects of Ultrathin Black Phosphorus on Hexagonal Boron Nitride. *IEEE Trans. Electron Devices* **64**, 5163–5171 (2017).

2. Datta, Supriyo. *Electronic Transport in Mesoscopic Systems*. (Cambridge University Press, 1995).

3. Penumatcha, Ashish V., Salazar, Ramon B. & Appenzeller, Joerg. Analysing black phosphorus transistors using an analytic Schottky barrier MOSFET model. *Nat. Commun.* **6**, 1–8 (2015).

4. Datta, Supriyo. *Quantum Transport Atom to Transistor*. (Cambridge University Press, 2005).

5. Wong, H. S. Philip & Akinwande, Deji. *Carbon Nanotube and Graphene Device Physics*. (Cambridge University Press, 2011).

6. Esqueda, Ivan S., Cress, Cory D., Che, Yuchi, Cao, Yu & Zhou, Chongwu. Charge trapping in aligned single-walled carbon nanotube arrays induced by ionizing radiation exposure. *J. Appl. Phys.* **115**, (2014).

7. Esqueda, Ivan S. & Barnaby, Hugh J. Modeling the non-uniform distribution of radiation-induced interface traps. *IEEE Trans. Nucl. Sci.* **59**, 723–727 (2012).
